# Survival outcomes of low-dose and high-dose bevacizumab front-line maintenance in advanced high-grade serous ovarian cancer: a propensity score-matched real-world study

**DOI:** 10.3389/fonc.2026.1890000

**Published:** 2026-07-01

**Authors:** Tao Wu, Yahui Shen, Xiaowei Wang, Ruxing Xi, Lijuan Hu, Jianrong Lu, Guoqing Wang, Pengchuang Zhang

**Affiliations:** 1 Department of Gynecologic Cancer, Shaanxi Provincial Cancer Hospital, Xi’an, Shaanxi, China; 2 Department of Pathology, Shaanxi Provincial Cancer Hospital, Xi’an, Shaanxi, China

**Keywords:** bevacizumab, dose, ovarian cancer, propensity score matching, survival, maintenance treatment

## Abstract

**Background:**

The optimal dose of bevacizumab for first-line maintenance therapy in advanced ovarian high-grade serous carcinoma (HGSC) remains controversial due to the lack of head-to-head RCTs. This real-world study aimed to compare the efficacy and safety of low-dose (7.5 mg/kg every 3 weeks) and high-dose (15 mg/kg every 3 weeks) bevacizumab in this population.

**Methods:**

We retrospectively analyzed patients with FIGO stage III–IV HGSC who received first-line bevacizumab maintenance therapy between January 2018 and May 2025. To control for confounding, 1:1 propensity score matching (PSM) was performed with sensitivity analyzes varying matching variables and caliper widths. Inverse probability of treatment weighting (IPTW) was used to confirm robustness. *Post-hoc* power analysis was performed The primary endpoint was progression-free survival (PFS); secondary endpoints were overall survival (OS) and safety.

**Results:**

Of 964 screened, 323 were eligible (low dose 179, high dose 144); after 1:1 PSM, 258 patients were matched (129/129) with standardized differences <0.1.After matching, PFS (HR 1.11, 95% CI 0.85–1.43, P = 0.450) and OS (HR 1.07, 95% CI 0.76–1.50, P = 0.688) did not differ significantly. IPTW analysis confirmed these findings (PFS: HR 1.06, P = 0.608; OS: HR 0.99, P = 0.942). *Post-hoc* power was 12% for the observed PFS HR of 1.11; the minimum detectable HR was 1.45 at 80% power. Prognostic analysis identified FIGO IV, non-complete resection (non-R0) residual disease, presence of ascites, BRCA-negative/unknown status, pre-maintenance CA125 ≥35 U/mL, and neoadjuvant chemotherapy followed by interval debulking surgery (NACT-IDS) as independent adverse factors; bevacizumab dose was not prognostic. Safety was comparable; proteinuria trended lower with low dose (6.98% vs 12.40%, *P* = 0.147), hypertension was similar, and no gastrointestinal perforations or treatment-related deaths occurred. Sensitivity analyzes were consistent.

**Conclusions:**

In patients with advanced ovarian HGSC, low-dose and high-dose bevacizumab first line maintenance showed no statistically significant difference in PFS or OS, with a favorable safety signal for the low dose regimen. However, the study was underpowered to confirm equivalence, and a clinically meaningful increase in progression risk cannot be excluded. These findings support the feasibility of low-dose bevacizumab as a first-line maintenance option, pending prospective validation.

## 1 Introduction

Ovarian cancer (OC) is a leading cause of gynecologic malignancy-related mortality worldwide, with high-grade serous carcinoma (HGSC) being the most common and aggressive histological subtype. The majority of patients are diagnosed at an advanced stage (FIGO III–IV), resulting in a five-year survival rate of 10% to 40% (1). Cytoreductive surgery combined with platinum and taxane-based chemotherapy remains the cornerstone of therapy for the advanced OC. Despite the disease’s initial sensitivity to chemotherapy, recurrence occurs in more than half of patients within two years, underscoring the critical unmet need for effective maintenance therapies to delay recurrence and improve long-term survival outcomes (2).

Bevacizumab inhibits tumor angiogenesis and has shown efficacy in phase III trials, improving progression-free survival (PFS) in advanced OC (3, 4). The landmark GOG-0218 study showed that bevacizumab at 15 mg/kg—given concurrently with chemotherapy and continued as maintenance—significantly prolonged PFS (5). Similarly, the ICON7 trial, using a lower dose of 7.5 mg/kg with the same chemotherapy backbone, also reported PFS benefits, particularly in high-risk patients (stage IV or sub-optimally debulked stage III) (6). However, no head-to-head RCT has compared these two regimens, leading to substantial global heterogeneity in real-world prescribing practices. The 15 mg/kg regimen predominates in Western countries, with 86% of patients in the French GINECO ENCOURAGE cohort receiving this dose (7). In contrast, the 7.5 mg/kg regimen is universally used in Serbia (8). Asian practices are equally divergent: Japanese trials (9)uniformly adopt 15 mg/kg, while an 8-year Taiwanese real-world study found most patients received <10 mg/kg per cycle (10). This practice variation, combined with the absence of direct comparative data, underscores the urgent need for robust real-world evidence to guide optimal dosing.

Emerging evidence from other clinical contexts further informs this dose-optimization question. In the recurrent OC setting, retrospective studies have demonstrated that a lower bevacizumab dose of 7.5 mg/kg can yield comparable PFS and OS compared to the standard 15 mg/kg regimen, while being associated with a lower incidence of hypertension (11, 12). The exploration of dose-efficacy relationships extends beyond OC. For instance, in glioblastoma, research suggests that adjusting the bevacizumab dose based on biomarker levels, such as VEGFA expression, could potentially improve clinical outcomes (13). These observations across different malignancies and treatment lines highlight a broader scientific interest in defining the minimal effective dose of bevacizumab to optimize the therapeutic index.

Consensus on the optimal first-line maintenance dose remains elusive. Recent network meta-analyzes suggest comparable efficacy between the two doses, with a more favorable safety profile for 7.5 mg/kg (14). However, these findings are limited by the inherent weaknesses of indirect comparisons. Furthermore, ethnic differences in drug metabolism, healthcare resource constraints, and reimbursement policies in China may alter the dose-response relationship, highlighting the critical need for population-specific real-world data.

To address this gap, we conducted a retrospective study of low-dose (7.5 mg/kg) vs. high-dose (15 mg/kg) bevacizumab in patients with advanced HGSC on first-line maintenance therapy. Using propensity score matching to adjust for baseline characteristics, we evaluated differences in PFS, overall survival (OS), and toxicity profiles. To our knowledge, this is the largest real-world study directly comparing these two regimens in the Chinese population. Our findings aim to inform clinical decision-making by providing real-world evidence on bevacizumab dosing optimization in this population.

## 2 Methods

### 2.1 Patients and study design

This study was approved by the Institutional Review Board of Shaanxi Provincial Cancer Hospital (No.2025-16), conducted in accordance with the Declaration of Helsinki. Written informed consent was waived due to the retrospective design. We enrolled consecutive patients with histologically confirmed FIGO stage III–IV HGSC who received first-line bevacizumab therapy at our center between January 2018 and May 2025. Mian inclusion criteria: ① Completed primary or interval debulking surgery followed by 4–8 cycles of platinum–taxane chemotherapy; ②Achieved complete/partial response (RECIST v1.1) to first-line chemotherapy; ③Initiated bevacizumab maintenance within 4–6 weeks post-chemotherapy at a fixed dose of 7.5 mg/kg q3w (low-dose) or 15 mg/kg q3w (high-dose); ④Follow-up duration ≥3 months. Main exclusion criteria: Non-HGSC histology, concurrent targeted therapy (PARP inhibitors/immunotherapy), or mixed bevacizumab doses; Disease progression during first-line chemotherapy or bevacizumab use in non-first-line settings. Clinical, pathological, and treatment data, including CA125 level (defined as the level at the last assessment prior to bevacizumab maintenance with a cutoff of ≥35 U/mL), were extracted from electronic medical records. A total of 964 patients met the inclusion criteria and were included in the initial cohort.

### 2.2 Outcome measures

The primary endpoint was maintenance progression-free survival (PFS), defined as the time from the initiation of bevacizumab maintenance therapy to the first documented disease progression or death from any cause, whichever occurred first. Disease progression was defined according to Response Evaluation Criteria in Solid Tumors (RECIST) version 1.1 guidelines (15), based on radiological, clinical, or symptomatic evidence, and did not include progression based solely on isolated CA-125 elevation. Patients alive and free of progression were censored at the time of their last disease evaluation. Overall survival (OS) was measured from the date of initiation of bevacizumab maintenance therapy to the date of death from any cause. Patients who experienced disease progression during chemotherapy were excluded from the analysis.

Treatment-related adverse events (AEs) were evaluated per the Common Terminology Criteria for Adverse Events (CTCAE v5.0) (16). Attribution to bevacizumab was determined by treating physicians. Safety analyzes included all-grade AEs, with particular attention to hypertension, proteinuria, bleeding, gastrointestinal perforation, and thromboembolic events. In addition to AE incidence, the clinical impact was assessed by reporting the proportion of patients requiring temporary interruption or permanent discontinuation of bevacizumab due to treatment-related AEs.

### 2.3 Statistical analysis

To minimize selection bias and balance baseline characteristics between the low-dose and high-dose groups, 1:1 nearest-neighbor propensity score matching (PSM) without replacement was performed (17). The primary analysis used a caliper width of 0.2 standard deviations of the logit of the propensity score. The propensity score was estimated using a logistic regression model that included age, FIGO stage, residual disease status after surgery, and BRCA mutation status. To assess the robustness of the PSM results, sensitivity analyzes were conducted using different variable selection criteria (all independent variables, variables with *P* < 0.05 in univariate analysis, variables with *P* < 0.2 in univariate analysis) and caliper widths (0.05, 0.1, and 0.2). As a complementary approach to address potential confounding, inverse probability of treatment weighting (IPTW) was applied to the entire cohort. Weights were estimated using logistic regression with logit link function and truncated at the 1st and 99th percentiles to reduce the influence of extreme weights. Balance after weighting was assessed using standardized mean differences, with SMD <0.1 considered indicative of adequate balance (18–20).

To quantify the minimum detectable effect size given the observed data, a *post-hoc* power calculation was performed using the Schoenfeld formula for the log-rank test (21). Based on the total number of PFS and OS events in the matched cohort, the minimum hazard ratio detectable with 80% power at a two-sided α of 0.05 was computed, and the *post-hoc* power for the observed HR in each endpoint was estimated. This analysis was conducted to inform the interpretation of non-significant survival comparisons and to quantify the risk of a type II error.

Associations between categorical variables were assessed with the chi-square or Fisher’s exact test. For time-to-event outcomes, Kaplan-Meier curves were plotted, and survival distributions were compared via the log-rank test. Univariate Cox proportional hazards regression was first performed to identify potential prognostic factors. Variables with a *P*-value < 0.2 in univariate analysis were entered into the multivariate Cox proportional hazards model, and no stepwise variable selection was applied. Independent prognostic factors for PFS and OS were identified through multivariate Cox proportional hazards models, which incorporated clinically relevant covariates. The findings from these models are presented as hazard ratios (HR) accompanied by 95% confidence intervals (CI).

All statistical analyzes were conducted using R software (version 4.3.3). The primary PSM and Cox regression analyzes were performed using the Zstats package (v1.0; www.zstats.net), an R-based analysis tool with published validation (22–24). Sensitivity analyzes, including IPTW, were conducted using standard R packages to verify result reproducibility. A two-sided p-value of less than 0.05 served as the threshold for statistical significance.

## 3 Results

### 3.1 Study population

**Figure 1:**
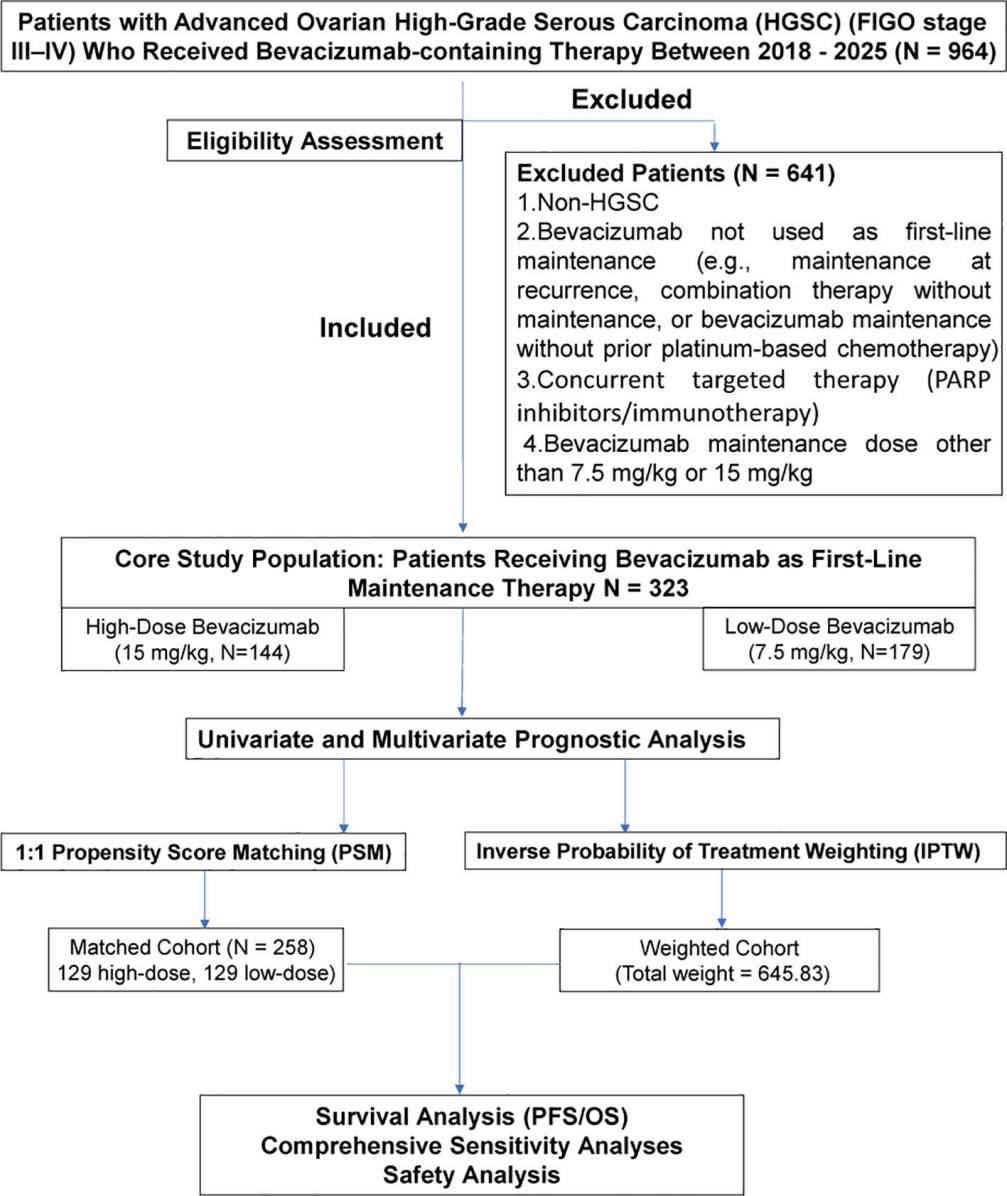
Patient selection and study design flowchart. This diagram illustrates the identification of patients with FIGO stage III–IV HGSC receiving first-line bevacizumab maintenance. Of 964 screened patients, 641 were excluded, leaving 323 eligible patients (179 low-dose, 144 high-dose). After 1:1 propensity score matching, 129 patients per group were included in the final analysis. inverse probability of treatment weighting (IPTW) was also applied as a complementary sensitivity approach. Final analyzes included survival assessment, comprehensive sensitivity analyzes, and safety evaluation. HGSC, high-grade serous carcinoma; FIGO, International Federation of Gynecology and Obstetrics; PSM, propensity score matching. IPTW, inverse probability of treatment weighting.

Based on the patient selection process outlined in **Figure 1**, a total of 964 consecutive patients with FIGO stage III–IV high-grade serous carcinoma (HGSC) who received bevacizumab-containing therapy at our institution between January 2018 and May 2025 were initially identified. After eligibility assessment, 641 patients were excluded from the final analysis due to non-HGSC histology, bevacizumab not used as first-line maintenance therapy (including maintenance administration at disease recurrence, combination chemotherapy without subsequent bevacizumab maintenance, or bevacizumab maintenance without prior platinum-based chemotherapy), or receipt of a non-standard bevacizumab maintenance dose other than 7.5 mg/kg or 15 mg/kg every 3 weeks. The final core study population therefore comprised 323 patients who received standard first-line bevacizumab maintenance therapy, including 179 patients treated with the low-dose regimen (7.5 mg/kg every 3 weeks) and 144 patients treated with the high-dose regimen (15 mg/kg every 3 weeks). The median number of bevacizumab cycles was 12 (IQR 9–15) in the overall cohort, with 12 (IQR 9–15) in the high-dose group and 11 (IQR 8–15) in the low-dose group (P = 0.100). The median cumulative bevacizumab dose was 180 mg/kg (IQR 135–225) in the high-dose group and 82.5 mg/kg (IQR 60–112.5) in the low-dose group. Among all 323 patients, the primary reasons for bevacizumab discontinuation were disease progression (42.0%), economic burden or insurance reimbursement limitations (26.0%), treatment-related adverse events (8.5%), and undocumented or unascertainable reasons (4.0%); the remaining patients either completed the planned maintenance course or were still on treatment at the data cutoff.

### 3.2 Baseline characteristics

The baseline characteristics of the 323 patients are summarized in **Table 1**. Prior to propensity score matching, significant imbalances were observed between the two groups. Patients in the high-dose bevacizumab group were more likely to have undergone neoadjuvant chemotherapy followed by interval debulking surgery (NACT-IDS) (64.6% vs. 48.6%, *P* = 0.004) and had a higher proportion of FIGO stage IV disease (29.2% vs. 19.0%, *P* = 0.032). No significant differences were found in age, ECOG performance status, residual disease status, BRCA mutation status, or other baseline variables between the two groups (all *P*>0.05).

**Table 1:** Baseline clinical and demographic characteristics of patients with FIGO stage III-IV high-grade serous ovarian cancer.

Variable	Total (n = 323)	High-dose (n = 144)	Low-dose (n = 179)	Statistic	P	SMD
BMI, M (Q_1_, Q_3_)	22.89 (21.87, 24.44)	22.72 (21.67, 24.34)	23.05 (21.99, 24.72)	Z=-1.372	0.170	0.140
CoB, M (Q_1_, Q_3_)	12.00 (9.00, 15.00)	12.00 (9.00, 15.00)	11.00 (8.00, 15.00)	Z=-1.645	0.100	-0.212
Age, n (%)				χ²=0.253	0.615	
<60	206 (63.78)	94 (65.28)	112 (62.57)			-0.056
≥60	117 (36.22)	50 (34.72)	67 (37.43)			0.056
ECOG, n (%)				χ²=0.046	0.830	
0	253 (78.33)	112 (77.78)	141 (78.77)			0.024
1	70 (21.67)	32 (22.22)	38 (21.23)			-0.024
Diabetes, n (%)				χ²=0.046	0.830	
No	253 (78.33)	112 (77.78)	141 (78.77)			0.024
Yes	70 (21.67)	32 (22.22)	38 (21.23)			-0.024
Treatment, n (%)				χ²=8.260	0.004	
PDS	143 (44.27)	51 (35.42)	92 (51.40)			0.320
NACT-IDS	180 (55.73)	93 (64.58)	87 (48.60)			-0.320
FIGO Stage, n (%)				χ²=4.589	0.032	
III	247 (76.47)	102 (70.83)	145 (81.01)			0.259
IV	76 (23.53)	42 (29.17)	34 (18.99)			-0.259
Residual disease, n (%)				χ²=0.636	0.425	
R0	138 (42.72)	58 (40.28)	80 (44.69)			0.089
Non-R0	185 (57.28)	86 (59.72)	99 (55.31)			-0.089
Ascites, n (%)				χ²=0.644	0.422	
Yes	174 (53.87)	74 (51.39)	100 (55.87)			0.090
No	149 (46.13)	70 (48.61)	79 (44.13)			-0.090
BRCA, n (%)				χ²=3.771	0.152	
Positive	59 (18.27)	33 (22.92)	26 (14.53)			-0.238
Negative	180 (55.73)	76 (52.78)	104 (58.10)			0.108
Unknown	84 (26.01)	35 (24.31)	49 (27.37)			0.069
CA125 n (%)				χ²=0.072	0.788	
<35 U/ml	200 (61.92)	88 (61.11)	112 (62.57)			0.030
≥35 U/ml	123 (38.08)	56 (38.89)	67 (37.43)			-0.030

BMI, Body Mass Index; CoB, Cycle of Bevacizumab; ECOG, Eastern Cooperative Oncology Group; PDS, Primary Debulking Surgery; NACT-IDS, Neoadjuvant Chemotherapy followed by Interval Debulking Surgery; FIGO, International Federation of Gynecology and Obstetrics; R0, No Gross Residual Disease; NED, No Evidence of Disease; CR, Complete Response; PR, Partial Response; CA125, Cancer Antigen 125; PSM, Propensity Score Matching; M (Q_1_, Q_3_), Median (Interquartile Range); SMD, Standardized Mean Difference.

Statistically significant differences (P<0.05) are indicated in bold. SMD < 0.1 was considered a good balance between groups. Bold P-values indicate significant between-group differences in demographic and clinical variables.

### 3.3 Prognostic factors for PFS and OS

Univariate and multivariate Cox regression analyzes for PFS and OS are summarized in **Tables 2**, **3**.

**Table 2:** Univariate and multivariate cox proportional hazards regression analysis of progression-free survival (PFS).

Variables	Univariate analysis					Multivariate analysis				
	β	S. E	*Z*	*P*	HR (95%CI)	β	S. E	*Z*	*P*	HR (95%CI)
Age										
<60					1.00 (Reference)					
≥60	0.03	0.12	0.26	0.792	1.03 (0.81 ~ 1.32)					
ECOG										
0					1.00 (Reference)					
1	0.16	0.15	1.11	0.265	1.18 (0.88 ~ 1.56)					
Diabetes										
No					1.00 (Reference)					
Yes	0.02	0.14	-0.14	0.888	0.98 (0.74 ~ 1.30)					
Surgical Treatment										
PDS					1.00 (Reference)					
NACT-IDS	0.11	0.12	0.96	0.335	1.12 (0.89 ~ 1.42)					
FIGO Stage										
III					1.00 (Reference)					1.00 (Reference)
IV	0.33	0.14	2.38	**0.018**	1.40 (1.06 ~ 1.84)	-0.04	0.15	-0.28	0.780	0.96 (0.72 ~ 1.28)
Residual disease										
R0					1.00 (Reference)					1.00 (Reference)
Non-R0	1.46	0.13	11.00	**<.001**	4.31 (3.32 ~ 5.59)	1.39	0.15	9.38	**<.001**	4.01 (3.00 ~ 5.36)
Ascites										
No					1.00 (Reference)					1.00 (Reference)
Yes	0.83	0.12	6.81	**<.001**	2.29 (1.81 ~ 2.91)	0.58	0.13	4.30	**<.001**	1.78 (1.37 ~ 2.31)
BRCA Status										
Positive					1.00 (Reference)					1.00 (Reference)
Negative	1.26	0.18	7.00	**<.001**	3.53 (2.48 ~ 5.02)	1.31	0.19	6.90	**<.001**	3.70 (2.55 ~ 5.36)
Unknown	1.12	0.20	5.66	**<.001**	3.07 (2.08 ~ 4.52)	1.09	0.21	5.25	**<.001**	2.96 (1.97 ~ 4.44)
CA125 Level										
<35 U/ml					1.00 (Reference)					1.00 (Reference)
≥35 U/ml	0.63	0.12	5.18	**<.001**	1.88 (1.48 ~ 2.39)	0.27	0.13	2.08	**0.038**	1.31 (1.02 ~ 1.68)
Bev. dose										
High					1.00 (Reference)					1.00 (Reference)
Low	0.22	0.12	1.81	0.060	1.24 (0.98 ~ 1.57)	0.34	0.12	2.77	**0.006**	1.41 (1.11 ~ 1.80)

β, regression coefficient; S.E., standard error; Z, Z-score; P, P-value; HR, hazard ratio; CI, confidence interval; ECOG, Eastern Cooperative Oncology Group; PDS, primary debulking surgery; NACT-IDS, neoadjuvant chemotherapy followed by interval debulking surgery; FIGO, International Federation of Gynecology and Obstetrics; R0, no gross residual disease after surgery; BRCA, breast cancer gene; CA125, cancer antigen 125; Bev., bevacizumab.

**Table 3:** Univariate and multivariate cox proportional hazards regression analysis of overall survival (OS).

Variables	Univariate analysis					Multivariate analysis				
	β	S. E	*Z*	*P*	HR (95%CI)	β	S. E	*Z*	*P*	HR (95%CI)
Age										
<60					1.00 (Reference)					
≥60	-0.15	0.17	-0.90	0.366	0.86 (0.62 ~ 1.19)					
ECOG										
0					1.00 (Reference)					
1	0.18	0.19	0.94	0.349	1.19 (0.83 ~ 1.72)					
Diabetes										
No					1.00 (Reference)					
Yes	0.01	0.19	0.08	0.940	1.01 (0.70 ~ 1.47)					
Surgical Treatment										
PDS					1.00 (Reference)					1.00 (Reference)
NACT-IDS	0.47	0.16	2.90	**0.004**	1.60 (1.16 ~ 2.19)	-0.32	0.19	-1.70	0.090	0.73 (0.50 ~ 1.05)
FIGO Stage										
III					1.00 (Reference)					1.00 (Reference)
IV	1.11	0.17	6.68	**<.001**	3.04 (2.19 ~ 4.21)	0.80	0.19	4.18	**<.001**	2.22 (1.53 ~ 3.22)
Residual disease										
R0					1.00 (Reference)					1.00 (Reference)
Non-R0	1.79	0.19	9.23	**<.001**	5.99 (4.09 ~ 8.76)	1.60	0.22	7.13	**<.001**	4.96 (3.19 ~ 7.70)
Ascites										
No					1.00 (Reference)					1.00 (Reference)
Yes	1.18	0.16	7.27	**<.001**	3.26 (2.37 ~ 4.48)	0.85	0.18	4.76	**<.001**	2.33 (1.65 ~ 3.30)
BRCA Status										
Positive					1.00 (Reference)					1.00 (Reference)
Negative	2.01	0.33	6.17	**<.001**	7.44 (3.93 ~ 14.08)	2.08	0.34	6.13	**<.001**	7.97 (4.10 ~ 15.49)
Unknown	1.79	0.34	5.22	**<.001**	5.99 (3.06 ~ 11.74)	1.73	0.35	4.94	**<.001**	5.64 (2.84 ~ 11.22)
CA125 Level										
<35 U/ml					1.00 (Reference)					1.00 (Reference)
≥35 U/ml	0.82	0.16	5.22	**<.001**	2.27 (1.67 ~ 3.08)	0.12	0.17	0.71	0.476	1.13 (0.81 ~ 1.57)
Bev. dose										
High					1.00 (Reference)					
Low	0.07	0.16	0.43	0.670	1.07 (0.79 ~ 1.46)					

β, regression coefficient; S.E., standard error; Z, Z-score; P, P-value; HR, hazard ratio; CI, confidence interval; ECOG, Eastern Cooperative Oncology Group; PDS, primary debulking surgery; NACT-IDS, neoadjuvant chemotherapy followed by interval debulking surgery; FIGO, International Federation of Gynecology and Obstetrics; R0, no gross residual disease after surgery; BRCA, breast cancer gene; CA125, cancer antigen 125; Bev., bevacizumab.

Bold P-values denote significant associations with progression-free survival or overall survival.

For PFS, univariate analysis identified FIGO stage IV, non-R0 residual disease, presence of ascites, BRCA mutation-negative/unknown status, CA125 ≥35 U/ml as significant adverse prognostic factors (all *P* < 0.05). Bevacizumab dose showed a trend toward inferior PFS in the low-dose group but did not reach statistical significance in univariate analysis (HR = 1.24, 95%CI 0.98-1.57, *P* = 0.060). However, after adjusting for confounding variables in multivariate analysis, low-dose bevacizumab emerged as an independent risk factor for worse PFS (HR = 1.41, 95%CI 1.11-1.80, *P* = 0.006). Given the observed baseline imbalances between the two groups and the discrepancy between univariate and multivariate analyzes, we further performed 1:1 propensity score matching (PSM) to minimize selection bias and rigorously evaluate the prognostic impact of different bevacizumab maintenance doses.

For OS, univariate analysis identified NACT-IDS, FIGO stage IV, non-R0 residual disease, presence of ascites, BRCA mutation-negative/unknown status, and CA125 ≥35 U/ml as significant predictors of poorer survival (all *P* < 0.05). No significant difference in OS was observed between the low-dose and high-dose groups in either univariate (HR = 1.07, *P* = 0.670) or multivariate analysis. Bevacizumab dose was not an independent prognostic factor for OS.

### 3.4 Survival outcomes after propensity score matching

The median follow-up for the entire cohort was 36.1 months (95% CI 32.6–39.6), ranging from 5.0 to 83.0 months. Before propensity score matching (n=323), the median PFS was 18.5 months (IQR 14.0–30.3) in the high-dose group and 16.0 months (IQR 11.0–26.5) in the low-dose group; the median OS was 37.0 months (IQR 30.8–48.0) versus 38.0 months (IQR 28.0–52.0), respectively. After 1:1 PSM (n=258), the median PFS was 17.0 months (IQR 13.0–26.0) in the high-dose group and 15.0 months (IQR 11.0–28.0) in the low-dose group; the median OS was 36.0 months (IQR 29.0–48.0) versus 38.0 months (IQR 29.0–51.0), respectively.

To address baseline imbalances between the two groups, 1:1 nearest-neighbor propensity score matching was performed using variables with *P* < 0.2 in univariate analysis and a caliper width of 0.2. After matching, 258 patients (129 in each group) were included in the final analysis. As shown in **Supplementary Table 1**, all baseline characteristics were well balanced between the low-dose and high-dose bevacizumab groups, with no statistically significant differences observed in any variable (all *P*>0.05) and all standardized mean differences (SMD) <0.1, indicating excellent matching quality.

**Figure 2:**
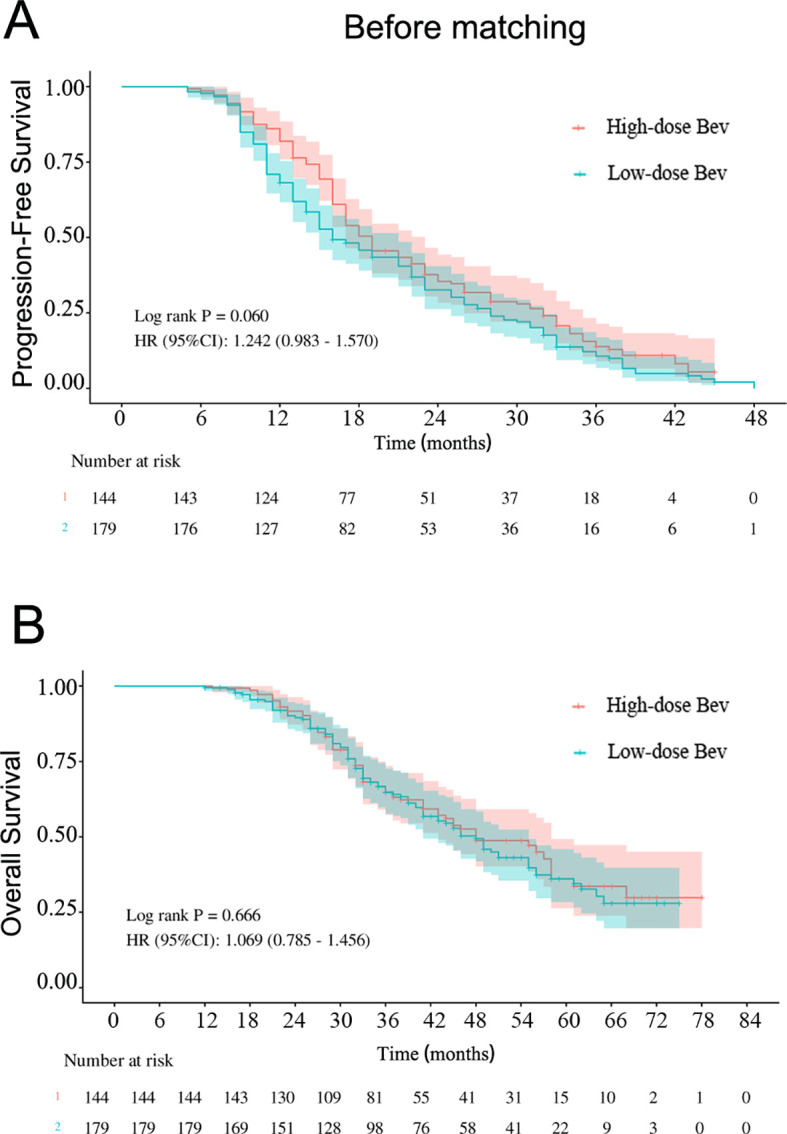
Kaplan-Meier survival curves before propensity score matching. **(A)** Progression-free survival (PFS) and **(B)** overall survival (OS) for patients receiving low-dose (7.5 mg/kg) versus high-dose (15 mg/kg) bevacizumab in the full cohort (n=323). Bev, bevacizumab; HR, hazard ratio; CI, confidence interval.

**Figure 3:**
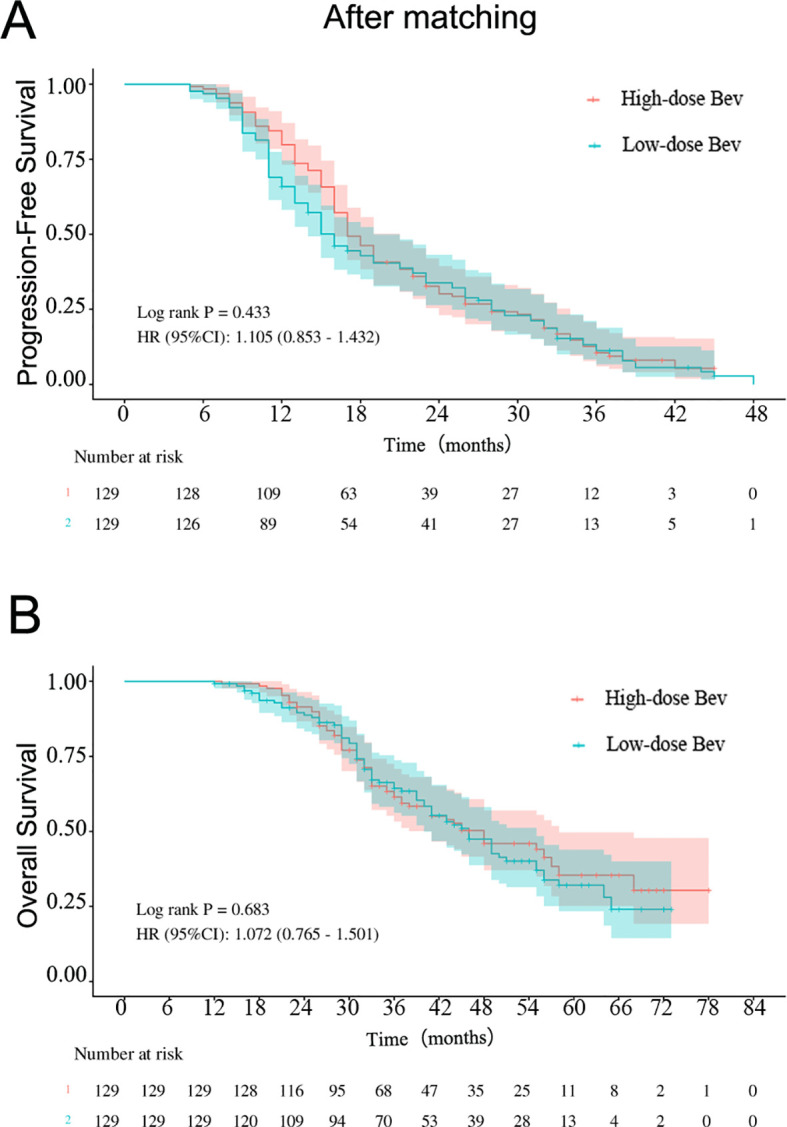
Kaplan-Meier survival curves after 1:1 propensity score matching. **(A)** Progression-free survival (PFS) and **(B)** overall survival (OS) for patients receiving low-dose (7.5 mg/kg) versus high-dose (15 mg/kg) bevacizumab in the matched cohort (n=258). Bev, bevacizumab; HR, hazard ratio; CI, confidence interval.

**Figure 2** presents Kaplan–Meier survival curves for the unmatched cohort. Before matching, there was a trend toward inferior PFS in the low-dose bevacizumab group, although this difference did not reach statistical significance (log-rank *P* = 0.060, HR = 1.24, 95%CI 0.98-1.57) (**Figure 2A**). No significant difference in OS was observed between the two groups before matching (log-rank *P* = 0.666, HR = 1.07, 95%CI 0.79-1.46) (**Figure 2B**). After propensity score matching (**Figure 3**), the difference in PFS between the two groups was eliminated (log-rank *P* = 0.433, HR = 1.09, 95%CI 0.85-1.43) (**Figure 3A**), and OS remained similar between the low-dose and high-dose groups (log-rank *P* = 0.683, HR = 1.07, 95%CI 0.77-1.50) (**Figure 3B**).

Cox regression analysis of the matched cohort confirmed these findings (**Table 4**). For PFS, non-R0 residual disease, presence of ascites, BRCA mutation-negative/unknown status, pre-maintenance CA125 ≥35 U/mL, NACT-IDS, and FIGO stage IV remained independent adverse prognostic factors (all *P* < 0.05). Notably, after adjusting for all confounding variables through propensity score matching, there was no significant difference in PFS between the low-dose and high-dose bevacizumab groups (HR 1.11, 95%CI 0.85–1.43, *P* = 0.450). For OS, the same six variables were identified as independent prognostic factors (all *P* < 0.05), while bevacizumab dose was not associated with OS in the matched cohort (HR = 1.07, 95%CI 0.76-1.50, *P* = 0.688).

**Table 4:** Cox regression analysis of PFS and OS in the PSM cohort.

Variables	PFS					OS				
	β	S. E	*Z*	*P*	HR (95%CI)	β	S. E	*Z*	*P*	HR (95%CI)
Age										
<60					1.00 (Reference)					1.00 (Reference)
≥60	0.05	0.14	0.39	0.699	1.05 (0.81 ~ 1.38)	-0.18	0.18	-0.97	0.332	0.84 (0.58 ~ 1.20)
ECOG										
0					1.00 (Reference)					1.00 (Reference)
1	0.06	0.16	0.36	0.716	1.06 (0.77 ~ 1.46)	0.10	0.21	0.46	0.648	1.10 (0.73 ~ 1.67)
Diabetes										
No					1.00 (Reference)					1.00 (Reference)
Yes	-0.05	0.16	-0.33	0.743	0.95 (0.70 ~ 1.29)	0.05	0.20	0.24	0.808	1.05 (0.70 ~ 1.57)
Surgical Treatment										
PDS					1.00 (Reference)					1.00 (Reference)
NACT-IDS	0.27	0.13	2.05	**0.041**	1.32 (1.01 ~ 1.71)	0.60	0.18	3.28	0.010	1.82 (1.27 ~ 2.61)
FIGO Stage										
III					1.00 (Reference)					1.00 (Reference)
IV	0.51	0.15	3.29	**<.001**	1.66 (1.23 ~ 2.25)	1.40	0.18	7.59	**<.001**	4.07 (2.83 ~ 5.85)
Residual disease										
R0					1.00 (Reference)					1.00 (Reference)
Non-R0	1.42	0.15	9.51	**<.001**	4.14 (3.09 ~ 5.55)	1.90	0.23	8.32	**<.001**	6.66 (4.26 ~ 10.42)
Ascites										
No					1.00 (Reference)					1.00 (Reference)
Yes	0.82	0.14	6.07	**<.001**	2.28 (1.75 ~ 2.97)	1.18	0.18	6.53	**<.001**	3.27 (2.29 ~ 4.66)
BRCA Status										
Positive					1.00 (Reference)					1.00 (Reference)
Negative	1.24	0.21	5.92	**<.001**	3.47 (2.30 ~ 5.23)	2.38	0.43	5.51	**<.001**	10.84 (4.65 ~ 25.29)
Unknown	1.11	0.23	4.80	**<.001**	3.04 (1.93 ~ 4.80)	2.09	0.45	4.63	**<.001**	8.05 (3.33 ~ 19.45)
CA125 Level										
<35 U/ml					1.00 (Reference)					1.00 (Reference)
≥35 U/ml	0.61	0.14	4.49	**<.001**	1.84 (1.41 ~ 2.40)	0.79	0.17	4.60	**<.001**	2.21 (1.58 ~ 3.11)
Bev. dose										
High					1.00 (Reference)					1.00 (Reference)
Low	0.10	0.13	0.76	0.450	1.11 (0.85 ~ 1.43)	0.07	0.17	0.40	0.688	1.07 (0.76 ~ 1.50)

β, regression coefficient; S.E., standard error; Z, Z-score; P, P-value; HR, hazard ratio; CI, confidence interval; ECOG, Eastern Cooperative Oncology Group; PDS, primary debulking surgery; NACT-IDS, neoadjuvant chemotherapy followed by interval debulking surgery; FIGO, International Federation of Gynecology and Obstetrics; R0, no gross residual disease after surgery; BRCA, breast cancer gene; CA125, cancer antigen 125; Bev., bevacizumab.

Bold P-values denote significant associations with progression-free survival or overall survival.

### 3.5 *Post-hoc* power analysis

To determine the minimum effect size detectable with the available data, a *post-hoc* power analysis was performed based on the observed event counts in the propensity score-matched cohort. In this cohort of 258 patients, 231 PFS events and 136 OS events were recorded. At a two-sided significance level of 0.05 and 80% power, the study was capable of detecting a hazard ratio (HR) of ≥1.45 for PFS and ≥1.62 for OS (**Supplementary Table 2**). The *post-hoc* power to detect the actually observed HR of 1.11 for PFS was 12%, and the power for the observed HR of 1.07 for OS was 5.9%. These results indicate that while the study could reasonably exclude a very large detrimental effect of low-dose bevacizumab, it was underpowered to detect moderate but clinically meaningful differences in survival. Therefore, the non-significant PFS and OS findings should not be interpreted as evidence of equivalence between the two dose regimens.

### 3.6 Robustness validation via multiple sensitivity analyzes

. The robustness of the primary findings was evaluated using two complementary approaches. First, PSM was repeated across nine parameters combining three variable selection criteria (all variables, P < 0.05, P < 0.2) and three caliper widths (0.05, 0.1, 0.2). No statistically significant association between bevacizumab dose and PFS or OS was observed in any PSM scenario (all P > 0.05). Second, IPTW was applied to the full cohort (n = 323). After weighting, covariate balance was adequate (most SMD < 0.1; **Supplementary Table 3**), and results were consistent with the primary PSM analysis: PFS HR 1.06 (95% CI 0.84–1.34, P = 0.608); OS HR 0.99 (95% CI 0.72–1.36, P = 0.942). All sensitivity results are summarized in **Supplementary Table 4**. The convergent findings across these ten analytical configurations confirm that no statistically significant difference in survival was observed between the two dose groups after rigorous confounding control.

### 3.7 Safety analysis

Safety outcomes were assessed in the PSM cohort (n=258) and are summarized in **Table 5**. Overall, any-grade adverse events occurred in 63.57% of patients, with grade 3–4 events in only 3.49%. Treatment interruption due to adverse events was required in 21.71% of the high-dose group versus 14.73% of the low-dose group; permanent discontinuation occurred in 10.85% versus 6.20%, respectively. No gastrointestinal perforation, fistula, or treatment-related death occurred in either group. Bevacizumab-specific toxicities were generally similar between groups, including hypertension (20.9% vs 18.6%, *P* = 0.869) and grade ≥3 hypertension (3.88% vs 3.10%). A trend toward lower proteinuria was noted in the low-dose group (6.98% vs 12.40%, *P* = 0.147). Bleeding and thromboembolic events were also balanced between groups. Chemotherapy-related adverse events, including neutropenia and thrombocytopenia, were similar in both arms. No treatment-related deaths were observed.

**Table 5:** Comparison of adverse events between high- and low-dose bevacizumab groups after 1:1 propensity score matching.

Variables	Total (n = 258)	High-dose (n = 129)	Low-dose (n = 129)	Statistic	P
Overall adverse events					
Any grade	164 (63.57)	77 (59.69)	87 (67.44)	χ²=1.356	0.244
Grade 3–4	9 (3.49)	6 (4.65)	3 (2.33)	χ²=0.461	0.497
Bleeding, n (%)				χ²=0.33	0.566
None	227 (87.98)	112 (86.82)	115 (89.15)		
Grade 1 or 2	31 (12.02)	17 (13.18)	14 (10.85)		
Hypertension, n (%)				-	0.869
None	207 (80.23)	102 (79.07)	105 (81.40)		
Grade 1 or 2	42 (16.28)	22 (17.05)	20 (15.50)		
Grade ≥3	9 (3.49)	5 (3.88)	4 (3.10)		
Proteinuria, n (%)				-	0.147
None	232 (89.92)	112 (86.82)	120 (93.02)		
Grade 1 or 2	25 (9.69)	16 (12.40)	9 (6.98)		
Grade 3	1 (0.39)	1 (0.78)	0 (0.00)		
Thromboembolic Events, n (%)				χ²=2.17	0.141
None	233 (90.31)	120 (93.02)	113 (87.60)		
Grade 1 or 2	25 (9.69)	9 (6.98)	16 (12.40)		
Neutropenia, n (%)				-	0.827
None	192 (74.42)	98 (75.97)	94 (72.87)		
Grade 1 or 2	57 (22.09)	27 (20.93)	30 (23.26)		
Grade ≥3	9 (3.49)	4 (3.10)	5 (3.88)		
Thrombocytopenia, n (%)				χ²=2.63	0.268
None	207 (80.23)	105 (81.40)	102 (79.07)		
Grade 1 or 2	39 (15.12)	16 (12.40)	23 (17.83)		
Grade ≥3	12 (4.65)	8 (6.20)	4 (3.10)		
Treatment modifications (due to adverse events)					
Treatment interruption	47 (18.22)	28 (21.71)	19 (14.73)	χ²=2.06	0.151
Permanent discontinuation	22 (8.53)	14 (10.85)	8 (6.20)	-	0.178

All adverse events were graded according to the Common Terminology Criteria for Adverse Events (CTCAE) version 5.0. Overall adverse events are calculated at the patient level: patients with multiple types of adverse events are counted only once in the “Any grade” and “Grade 3–4” summary rows. χ²: Chi-square test; -: Fisher exact test.

No grade ≥4 non-hematologic adverse events, gastrointestinal perforation, or treatment-related deaths were observed in either group.

## 4 Discussion

Our large real-world study provides robust evidence on the dose–efficacy relationship of bevacizumab in Chinese patients with advanced HGSC. After 1:1 PSM, no statistically significant difference in PFS (HR 1.11, 95% CI 0.85–1.43, P = 0.450) or OS (HR 1.07, 95% CI 0.76–1.50, P = 0.688) was observed between low-dose (7.5 mg/kg) and high-dose (15 mg/kg) groups. Notably, the low-dose regimen was associated with a favorable safety profile, including a trend toward lower proteinuria and numerically fewer treatment interruptions and permanent discontinuations. No gastrointestinal perforation or treatment-related deaths occurred in either cohort.

### 4.1 Interpretation of non-significant findings

It is important to emphasize that a non-significant P value does not constitute evidence of equivalence. The 95% confidence interval for PFS (0.85–1.43) is compatible with up to a 43% increase in the risk of progression with low-dose bevacizumab—a magnitude that could be clinically meaningful. Our *post-hoc* power analysis, based on 231 PFS events, showed that the study had 80% power to detect an HR of only ≥1.45, and the power for the observed HR of 1.11 was 12%. For OS (136 events), the minimum detectable HR was 1.62, with 5.9% power. These results indicate that while a large detrimental effect of low-dose bevacizumab can be reasonably excluded, moderate but clinically relevant inferiority cannot be ruled out. Therefore, our findings should not be interpreted as demonstrating equivalence, but rather as suggesting that any true survival difference between the two doses is unlikely to be extremely large.

### 4.2 Relationship to existing evidence

Our results are consistent with the growing body of real-world evidence on this question. The recent network meta-analysis by Ethier et al (14) reported overlapping efficacy estimates between the two doses in indirect comparisons and a better safety profile for the low-dose regimen. East Asian real-world observations: the Japanese JGOG3022 trial (9)and Taiwanese ROBOT study (10) have documented divergent bevacizumab dosing practices in Asian populations, while our large Chinese cohort provides the largest direct real-world comparison to date. These converging data raise the hypothesis that the dose-response relationship for bevacizumab in ovarian cancer may plateau above a certain threshold, and that tumor biology and surgical factors exert a far greater influence on prognosis than maintenance dose intensity. Multivariate analysis confirmed BRCA mutation status as a strong independent prognostic factor for both PFS and OS, consistent with prior landmark trials. However, whether the efficacy of different bevacizumab doses varies according to BRCA or other molecular subtypes could not be formally assessed in this study, as no subgroup interaction analysis was performed. Dedicated biomarker-stratified prospective trials are needed to address this question (25–27).

### 4.3 Reconciling the pre-matching and post-matching PFS discrepancy

Before matching, a conventional multivariate Cox model suggested that low-dose bevacizumab was associated with worse PFS (HR 1.41, P = 0.006). This signal completely resolved after PSM and IPTW, both of which achieved superior covariate balance. The discrepancy is attributable to the pronounced baseline imbalance in FIGO stage and surgical approach: the high-dose group was enriched with stage IV disease and NACT-IDS patients. Although these covariates were included in the regression model, standard adjustment could not fully eliminate residual confounding (28). The consistent null findings across ten different analytical configurations (nine PSM specifications and IPTW) confirm that the initial signal was spurious. This underscores the value of propensity score-based methods when treatment groups differ substantially in baseline risk.

### 4.4 Safety profile and clinical implications

The safety analysis, expanded during revision to include treatment modification data, demonstrated that both regimens were well tolerated. Any-grade adverse events occurred in 63.57% of patients, with grade 3–4 events in only 3.49%. Grade ≥3 hypertension rates (high-dose 3.88% vs. low-dose 3.10%) compared favorably with those reported in GOG-0218 (10.5%) and ICON7 (6%). Proteinuria trended lower with low-dose (6.98% vs. 12.40%), consistent with the dose-dependent nature of VEGF inhibition-related renal effects. Treatment interruption (21.71% vs. 14.73%) and permanent discontinuation (10.85% vs. 6.20%) were numerically lower in the low-dose group. Although these differences did not reach statistical significance, the consistently favorable trends support the clinical feasibility of the low-dose regimen for maintenance therapy, where prolonged exposure amplifies the cumulative burden of even low-grade toxicities on quality of life and adherence.

### 4.5 Maintenance duration in real-world context

The median bevacizumab maintenance duration in our cohort (12 cycles) was shorter than the protocol-specified durations in GOG-0218 and ICON7. However, as summarized in **Supplementary Table 5**, this is consistent with real-world experience globally: median cycles ranged from 7.9 (Taiwan ROBOT) (10) to 18 (France ENCOURAGE) (7), with Japan JGOG3022 (9) reporting 17, Austria (29) 15, and Canada (30) a mean of 12 cycles. Disease progression was the dominant reason for early discontinuation in our cohort (42.0%), comparable to rates reported in ENCOURAGE (37%) and JGOG3022 (26.6%). Toxicity-related discontinuation was low (8.5%), comparing favorably with JGOG3022 (14.0%). Notably, 26.0% of patients discontinued due to economic burden—a barrier also prominent in the Taiwanese ROBOT study (10). This directly underscores the relevance of our dose-comparison question: in settings where financial constraints limit treatment duration, identifying the lowest effective dose is clinically important. The abbreviated maintenance in both arms may also attenuate any true dose-dependent survival difference, further supporting our conservative interpretation.

Regarding the initial dose selection, our institution had no formal protocol mandating a specific bevacizumab dose during the study period. As shown in **Table 1**, patients in the high-dose group had significantly higher proportions of FIGO stage IV disease and NACT-IDS, suggesting that physicians tended to select the 15 mg/kg dose for patients perceived to be at greater risk of recurrence. Economic considerations also influenced prescribing: the 50% lower per-cycle cost of the 7.5 mg/kg regimen made it a practical choice for patients with limited insurance coverage or financial constraints. This real-world dose selection pattern reflects the very practice variation that motivated our study.

### 4.6 Exclusion of PARP inhibitor recipients and clinical applicability

The present study focused exclusively on bevacizumab single-agent maintenance, deliberately excluding patients who received concomitant PARP inhibitors. This design aligns the study population with the treatment paradigm of the landmark GOG-0218 and ICON7 trials, in which bevacizumab was evaluated without PARP inhibitor maintenance. From a methodological standpoint, including PARP inhibitor recipients would have introduced a dominant confounder: biomarker-dependent survival benefit of PARP inhibitors is inextricably linked to BRCA/HRD status and would have obscured any marginal effect attributable to bevacizumab dose. Because the study population mirrors that of the pivotal trials, the findings are most directly generalizable to patients treated with bevacizumab monotherapy. This remains a clinically relevant population: current guidelines (NCCN, ESMO) recommend bevacizumab monotherapy as a standard first-line maintenance option for HRD-negative patients, for those who discontinue PARP inhibitors due to toxicity (approximately 15–20% in clinical practice) (31, 32), and in settings where PARP inhibitors are inaccessible due to cost. Whether the observed safety and efficacy patterns extend to combined bevacizumab–PARP inhibitor maintenance is an open question being prospectively evaluated in the PGOG-ov1 trial.

### 4.7 Strengths and limitations

This study offers several key contributions to the literature. It represents the largest real-world comparison of low-dose versus high-dose bevacizumab in patients with advanced HGSC, addressing a critical gap in regional clinical evidence. Rigorous confounding control was applied through PSM, IPTW, and multiple sensitivity analyzes, with results remaining consistent across all methods. The addition of *post-hoc* power analysis quantifies the limits of what can be concluded from our non-significant findings. Additionally, our safety data reinforce the clinical feasibility of low-dose bevacizumab, which may inform cost-effective and tolerable treatment strategies for Chinese patients.

Several limitations should be acknowledged. First, the retrospective design may introduce unmeasured confounding despite comprehensive sensitivity analyzes. Second, complete data on post-progression therapies were not available, which may influence OS outcomes (33). Third, although extensive sensitivity analyzes confirmed result stability, the sample size—although the largest direct comparison to date—was insufficient to exclude moderate differences in survival or to formally test treatment-by-biomarker interactions. Fifth, the exclusion of patients receiving PARP inhibitors limits direct generalizability to contemporary combined maintenance, although this was necessary to preserve internal validity.

## 5 Future perspectives

The ongoing PGOG-ov1 trial (34), a multicenter randomized phase III study directly comparing 7.5 mg/kg with 15 mg/kg bevacizumab with or without olaparib and stratified by BRCA/HRD status, is designed to definitively answer this question s. Our findings provide real-world evidence supporting the rationale for this trial. Future research should prioritize: (1) prospective validation of dose equivalence or non-inferiority; (2) identification of predictive biomarkers beyond BRCA/HRD, such as angiogenic signatures (35–37), may help refine patient selection and guide truly individualized dosing; (3) comprehensive health economic evaluations weighing the reduced drug costs and favorable toxicity profile of the 7.5 mg/kg regimen against the 15 mg/kg dose, particularly for resource-constrained healthcare settings (38, 39).

## 6 Conclusion

In this large real-world study of Chinese patients with advanced HGSC, no statistically significant difference in PFS or OS was observed between low-dose (7.5 mg/kg) and high-dose (15 mg/kg) bevacizumab maintenance after rigorous confounding control. The low-dose regimen was associated with a favorable safety profile. However, *post-hoc* power analysis demonstrated that the study was underpowered to confirm equivalence, and a clinically meaningful increase in progression risk cannot be excluded. These findings provide real-world evidence supporting the feasibility of low-dose bevacizumab for first-line maintenance therapy, while underscoring the need for prospective non-inferiority trials to definitively establish dose equivalence.

## Data Availability

The raw data supporting the conclusions of this article will be made available by the authors, without undue reservation.
