# Supplementary material for: Survival outcomes of low-dose and high-dose bevacizumab front-line maintenance in advanced high-grade serous ovarian cancer: a propensity score-matched real-world study
Source: Front Oncol. 2026 Jul 1;16:1890000. doi: 10.3389/fonc.2026.1890000 (PMC13368989; doi:10.3389/fonc.2026.1890000)
Supplement: Supplementary file 1 [file Table1.docx]

### Table S1. Baseline Characteristics After 1:1 Propensity Score Matching

| Variable | Total (n = 258) | High-Dose (n = 129) | Low-Dose (n = 129) | Statistic | *P* | SMD |
| --- | --- | --- | --- | --- | --- | --- |
|  |  |  |  |  |  |  |
| BMI, M (Q₁, Q₃) | 22.89 (21.87, 24.44) | 22.76 (21.78, 24.44) | 23.05 (21.91, 24.44) | Z=-0.562 | 0.574 | 0.079 |
| CoB, M (Q₁, Q₃) | 12.00 (9.00, 14.00) | 12.00 (9.00, 14.00) | 11.00 (8.00, 14.00) | Z=-0.690 | 0.490 | -0.091 |
| Age, n (%) |  |  |  | χ²=0.417 | 0.519 |  |
| ＜60 | 163 (63.18) | 84 (65.12) | 79 (61.24) |  |  | -0.080 |
| ≥60 | 95 (36.82) | 45 (34.88) | 50 (38.76) |  |  | 0.080 |
| ECOG, n (%) |  |  |  | χ²=1.132 | 0.287 |  |
| 0 | 203 (78.68) | 98 (75.97) | 105 (81.40) |  |  | 0.139 |
| 1 | 55 (21.32) | 31 (24.03) | 24 (18.60) |  |  | -0.139 |
| Diabetes, n (%) |  |  |  | χ²=0.022 | 0.882 |  |
| No | 199 (77.13) | 99 (76.74) | 100 (77.52) |  |  | 0.019 |
| Yes | 59 (22.87) | 30 (23.26) | 29 (22.48) |  |  | -0.019 |
| Treatment, n (%) |  |  |  | χ²=0.576 | 0.448 |  |
| PDS | 106 (41.09) | 50 (38.76) | 56 (43.41) |  |  | 0.094 |
| NACT-IDS | 152 (58.91) | 79 (61.24) | 73 (56.59) |  |  | -0.094 |
| FIGO Stage, n (%) |  |  |  | χ²=0.081 | 0.775 |  |
| III | 192 (74.42) | 95 (73.64) | 97 (75.19) |  |  | 0.036 |
| IV | 66 (25.58) | 34 (26.36) | 32 (24.81) |  |  | -0.036 |
| Residual disease, n (%) |  |  |  | χ²=0.580 | 0.446 |  |
| R0 | 104 (40.31) | 49 (37.98) | 55 (42.64) |  |  | 0.094 |
| Non-R0 | 154 (59.69) | 80 (62.02) | 74 (57.36) |  |  | -0.094 |
| Ascites, n (%) |  |  |  | χ²=0.388 | 0.533 |  |
| Yes | 135 (52.33) | 65 (50.39) | 70 (54.26) |  |  | 0.078 |
| No | 123 (47.67) | 64 (49.61) | 59 (45.74) |  |  | -0.078 |
| BRCA, n (%) |  |  |  | χ²=0.578 | 0.749 |  |
| Positive | 42 (16.28) | 22 (17.05) | 20 (15.50) |  |  | -0.043 |
| Negative | 150 (58.14) | 72 (55.81) | 78 (60.47) |  |  | 0.095 |
| Unknown | 66 (25.58) | 35 (27.13) | 31 (24.03) |  |  | -0.073 |
| CA125 n (%) |  |  |  | χ²=1.053 | 0.305 |  |
| ＜35 U/ml | 160 (62.02) | 76 (58.91) | 84 (65.12) |  |  | 0.130 |
| ≥35 U/ml | 98 (37.98) | 53 (41.09) | 45 (34.88) |  |  | -0.130 |

**Abbreviations**

BMI, Body Mass Index; CoB, Cycle of Bevacizumab; ECOG, Eastern Cooperative Oncology Group; PDS, Primary Debulking Surgery; NACT-IDS, Neoadjuvant Chemotherapy followed by Interval Debulking Surgery; FIGO, International Federation of Gynecology and Obstetrics; R0, No Gross Residual Disease; NED, No Evidence of Disease; CR, Complete Response; PR, Partial Response; CA125, Cancer Antigen 125; PSM, Propensity Score Matching; M (Q₁, Q₃), Median (Interquartile Range); SMD, Standardized Mean Difference.

Notes: Statistically significant differences (P<0.05) are indicated in bold. SMD < 0.1 was considered as good balance between groups.
