# Supplementary material for: Survival outcomes of low-dose and high-dose bevacizumab front-line maintenance in advanced high-grade serous ovarian cancer: a propensity score-matched real-world study
Source: Front Oncol. 2026 Jul 1;16:1890000. doi: 10.3389/fonc.2026.1890000 (PMC13368989; doi:10.3389/fonc.2026.1890000)
Supplement: Supplementary file 2 [file Table2.docx]

**Table S2**

**Table S2. Post-hoc Power Analysis for Progression-Free Survival and Overall Survival in the Propensity Score-Matched Cohort**

| Endpoint | Total Events | Observed HR (Low vs. High Dose) | Minimum Detectable HR at 80% Power | Post-hoc Power for Observed HR |
| --- | --- | --- | --- | --- |
| PFS | 231 | 1.11 | 1.45 | 12.0% |
| OS | 136 | 1.07 | 1.62 | 5.9% |

*Abbreviations:* PFS, progression-free survival; OS, overall survival; HR, hazard ratio.
*Notes:* All calculations used the Schoenfeld formula for the log-rank test (Schoenfeld, 1983; Collett, 2015). The minimum detectable HR was computed at a two-sided α of 0.05 with 80% power. The reference group is the high-dose bevacizumab group (15 mg/kg every 3 weeks). The observed HRs are derived from univariate Cox proportional hazards regression in the 1:1 propensity score-matched cohort (n = 258).

Reference:

Schoenfeld DA. Sample-size formula for the proportional-hazards regression model. Biometrics. 1983;39(2):499-503.

Collett D. Modelling Survival Data in Medical Research. 3rd ed. Boca Raton: CRC Press; 2015.
