# Supplementary material for: Survival outcomes of low-dose and high-dose bevacizumab front-line maintenance in advanced high-grade serous ovarian cancer: a propensity score-matched real-world study
Source: Front Oncol. 2026 Jul 1;16:1890000. doi: 10.3389/fonc.2026.1890000 (PMC13368989; doi:10.3389/fonc.2026.1890000)
Supplement: Supplementary file 3 [file Table3.docx]

### **Table S3. Baseline Characteristics After Inverse Probability of Treatment Weighting**

| Variable | Total | High-dose | Low-dose | Statistic | *P* | SMD |
| --- | --- | --- | --- | --- | --- | --- |
|  |  |  |  |  |  |  |
| N (weight) | 645.83 | 323.43 | 322.40 |  |  |  |
| BMI, M (Q₁, Q₃) | 22.94 (21.88,24.52) | 22.83 (21.78, 24.52) | 23.05 (21.88, 24.52) | Z=-0.22 | 0.829 | 0.017 |
| CoB, M (Q₁, Q₃) | 12.00 (9.00,15.00) | 12.00 (9.00, 15.00) | 12.00 (8.00, 15.00) | Z=-0.09 | 0.932 | 0.005 |
| Age, n (%) |  |  |  | χ²=0.56 | 0.470 | 0.083 |
| ＜60 | 418.43 (64.79) | 215.98 (66.78) | 202.44 (62.79) |  |  |  |
| ≥60 | 227.41 (35.21) | 107.45 (33.22) | 119.96 (37.21) |  |  |  |
| ECOG, n (%) |  |  |  | χ²=0.01 | 0.914 | 0.012 |
| 0 | 514.33 (79.64) | 258.36 (79.88) | 255.97 (79.40) |  |  |  |
| 1 | 131.50 (20.36) | 65.07 (20.12) | 66.43 (20.60) |  |  |  |
| Diabetes, n (%) |  |  |  | χ²=0.00 | 0.954 | 0.007 |
| No | 504.06 (78.05) | 252.88 (78.19) | 251.18 (77.91) |  |  |  |
| Yes | 141.77 (21.95) | 70.55 (21.81) | 71.22 (22.09) |  |  |  |
| Treatment, n (%) |  |  |  | χ²=0.00 | 0.983 | 0.003 |
| PDS | 286.65 (44.39) | 143.76 (44.45) | 142.90 (44.32) |  |  |  |
| NACT-IDS | 359.18 (55.61) | 179.68 (55.55) | 179.50 (55.68) |  |  |  |
| FIGO Stage, n (%) |  |  |  | χ²=0.00 | 0.958 | 0.006 |
| III | 491.53 (76.11) | 246.58 (76.24) | 244.95 (75.98) |  |  |  |
| IV | 154.31 (23.89) | 76.85 (23.76) | 77.45 (24.02) |  |  |  |
| Residual disease, n (%) |  |  |  | χ²=1.18 | 0.299 | 0.121 |
| R0 | 275.37 (42.64) | 128.24 (39.65) | 147.13 (45.63) |  |  |  |
| Non-R0 | 370.46 (57.36) | 195.19 (60.35) | 175.27 (54.37) |  |  |  |
| Ascites, n (%) |  |  |  | χ²=0.89 | 0.367 | 0.105 |
| Yes | 344.04 (53.27) | 163.86 (50.66) | 180.18 (55.89) |  |  |  |
| No | 301.79 (46.73) | 159.58 (49.34) | 142.22 (44.11) |  |  |  |
| BRCA, n (%) |  |  |  | χ²=0.01 | 0.998 | 0.008 |
| Positive | 115.68 (17.91) | 58.04 (17.94) | 57.65 (17.88) |  |  |  |
| Negative | 358.46 (55.50) | 179.99 (55.65) | 178.47 (55.36) |  |  |  |
| Unknown | 171.69 (26.58) | 85.40 (26.41) | 86.29 (26.76) |  |  |  |
| CA125, n (%) |  |  |  | χ²=0.22 | 0.649 | 0.053 |
| ＜35 U/ml | 402.18 (62.27) | 197.28 (61.00) | 204.90 (63.55) |  |  |  |
| ≥35 U/ml | 243.65 (37.73) | 126.15 (39.00) | 117.50 (36.45) |  |  |  |

**Abbreviations**

BMI, Body Mass Index; CoB, Cycle of Bevacizumab; ECOG, Eastern Cooperative Oncology Group; PDS, Primary Debulking Surgery; NACT-IDS, Neoadjuvant Chemotherapy followed by Interval Debulking Surgery; FIGO, International Federation of Gynecology and Obstetrics; R0, No Gross Residual Disease; NED, No Evidence of Disease; CR, Complete Response; PR, Partial Response; CA125, Cancer Antigen 125; PSM, Propensity Score Matching; M (Q₁, Q₃), Median (Interquartile Range); SMD, Standardized Mean Difference; Z: Mann-Whitney test, χ²: Chi-square test

Notes:

N (weight) represents the sum of inverse probability weights, i.e., the effective sample size after weighting, not the actual number of enrolled patients.

Continuous variables are presented as median (interquartile range), and categorical variables are presented as weighted number (weighted percentage).

Mann-Whitney U test was used for comparisons of continuous variables, and Chi-square test was used for comparisons of categorical variables.

Statistically significant differences (P<0.05) are indicated in bold.

Standardized mean difference (SMD) < 0.1 was considered as good baslance between groups.
