# Supplementary material for: Survival outcomes of low-dose and high-dose bevacizumab front-line maintenance in advanced high-grade serous ovarian cancer: a propensity score-matched real-world study
Source: Front Oncol. 2026 Jul 1;16:1890000. doi: 10.3389/fonc.2026.1890000 (PMC13368989; doi:10.3389/fonc.2026.1890000)
Supplement: Supplementary file 4 [file Table4.docx]

**Table S4. Sensitivity Analysis of Bevacizumab Maintenance Dose and Survival Outcomes Under Different Confounding Control Methods**

| **Confounding Control Method & Parameters** |  | **PFS** |  | **OS** |  |
| --- | --- | --- | --- | --- | --- |
| **1:1 Nearest Neighbor PSM** |  |  |  |  |  |
| Variable Selection | Caliper Value | HR (95% CI) | *P* | HR (95% CI) | *P* |
| All independent variables | 0.05 | 1.15 (0.88–1.51) | 0.314 | 1.04 (0.73–1.48) | 0.832 |
| Variables with P < 0.05 | 0.05 | 1.03 (0.78–1.36) | 0.839 | 1.02 (0.71–1.47) | 0.901 |
| Variables with P < 0.2 | 0.05 | 1.02 (0.77–1.34) | 0.912 | 1.05 (0.73–1.51) | 0.788 |
| All independent variables | 0.1 | 1.13 (0.87–1.47) | 0.375 | 1.05 (0.74–1.49) | 0.781 |
| Variables with P < 0.05 | 0.1 | 1.00 (0.77–1.31) | 0.988 | 1.00 (0.71–1.41) | 0.993 |
| Variables with P < 0.2 | 0.1 | 1.04 (0.80–1.37) | 0.756 | 1.06 (0.74–1.51) | 0.763 |
| All independent variables | 0.2 | 1.25 (0.96–1.61) | 0.095 | 1.11 (0.79–1.55) | 0.560 |
| Variables with P < 0.05 | 0.2 | 1.11 (0.86–1.43) | 0.425 | 0.98 (0.70–1.37) | 0.909 |
| Variables with P < 0.2 | 0.2 | 1.11 (0.85–1.43) | 0.450 | 1.07 (0.76–1.50) | 0.688 |
| **IPTW** |  |  |  |  |  |
| Full sample, ATE estimation |  | 1.06 (0.84–1.34) | 0.608 | 0.99 (0.72–1.36) | 0.942 |

Abbreviations:

PSM, propensity score matching; IPTW, inverse probability of treatment weighting; ATE, average treatment effect; HR, hazard ratio; CI, confidence interval; PFS, progression-free survival; OS, overall survival.

Notes:

All hazard ratios (HR) and 95% confidence intervals (CI) were calculated using univariate Cox proportional hazards regression.

The reference group was the high-dose bevacizumab group (15 mg/kg every 3 weeks); the low-dose group was 7.5 mg/kg every 3 weeks.

For propensity score matching analyses: 1:1 nearest neighbor matching was used. Caliper values are expressed as multiples of the standard deviation of the propensity score.

For inverse probability of treatment weighting analysis: The full cohort of 323 patients was included. Weights were estimated using logistic regression with logit link function, and truncated at the 1st and 99th percentiles to reduce the influence of extreme weights.

Post-hoc power for all sensitivity analyses was uniformly low, consistent with the primary analysis (see Table S2)
