# Supplementary material for: Survival outcomes of low-dose and high-dose bevacizumab front-line maintenance in advanced high-grade serous ovarian cancer: a propensity score-matched real-world study
Source: Front Oncol. 2026 Jul 1;16:1890000. doi: 10.3389/fonc.2026.1890000 (PMC13368989; doi:10.3389/fonc.2026.1890000)
Supplement: Supplementary file 5 [file Table5.docx]

**Table S5. Global Real-World Comparison of Bevacizumab Front-line Maintenance Treatment Duration in Advanced Ovarian Cancer**

| **Study Region/Name** | **Publication Year** | **Sample Size** | **Median Total Bevacizumab Cycles (IQR/Range)** | **Primary Reasons for Discontinuation** |
| --- | --- | --- | --- | --- |
| France ENCOURAGE cohort (7) | 2021 | 468 | 18 (10–21) | 37% disease progression, 8% toxicity |
| Japan JGOG3022 (9) | 2019 | 293 | 17 (1–40) | 26.6% primary disease progression, 14.0% serious adverse events, 5.5% patient refusal due to adverse events, 3.8% patient refusal for other reasons; 15.0% still receiving bevacizumab at cutoff |
| Austria (29) | 2022 | 50 | 15 (1–30) | 66% (33/50) experienced treatment interruptions due to adverse events or planned surgical procedures |
| Canada (30) | 2023 | 282 | 12 (mean 10, 1–17) | Reasons for early discontinuation cannot be ascertained from administrative health databases |
| Present Study (Mainland China) | 2025 | 323 | 12 (9–15) | 42.0% disease progression, 26.0% economic burden and insurance reimbursement limits, 8.5% treatment-related adverse events, 4.0% undocumented/unascertainable reasons |
| Taiwan, China ROBOT (10) | 2020 | 77 | 7.9 (1–17) | Not explicitly reported |
